# Trichomes as a defense mechanism against climbing plants

**DOI:** 10.1080/15592324.2025.2548303

**Published:** 2025-08-22

**Authors:** Pavol Prokop

**Affiliations:** a Department of Environmental Ecology and Landscape Management, Faculty of Natural Sciences, Comenius University, Bratislava, Slovakia; b Institute of Zoology, Slovak Academy of Sciences, Bratislava, Slovakia

**Keywords:** Thigmotropism, twinning plants, interspecific competition

## Abstract

Non-glandular trichomes are essential in plant defence against herbivores and water loss. However, evolutionary pressures often favor the development of multifunctional traits, suggesting that trichomes may serve multiple ecological roles. I hypothesized that the stem trichomes of *Anchusa officinalis* may reduce the likelihood of climbing plants attaching to them, thereby limiting competition for pollinators, light, water, and nutrients. Field observations showed that the majority (96%) of *Convolvulus arvensis*, a common climbing weed that co-occurs with *A. officinalis*, did not coil its stems. Pot experiments supported these findings: *C. arvensis* coiled around *A. officinalis* less frequently, more slowly (measured as time to first coil), and less intensely (measured by the number of coils) compared to plants with artificial soft trichomes (wire rods with soft plastic bristles), wooden sticks, or *A. officinalis* with trichomes removed. In contrast, *C. arvensis* coiled around hard artificial trichomes at similarly low rates as it did around the *A. officinalis* treatment. While biochemical factors could potentially explain the reduced interaction, it seems less likely, given that the main function of non-glandular trichomes in this species is water conservation. This role typically does not involve the secretion of specific chemicals. I suggest that mechanical stress caused by trichomes may trigger internal signaling in *C. arvensis*, altering its climbing behavior through thigmotropic responses.

## Introduction

Plants possess both glandular and non-glandular trichomes, which are hair-like surface structures with distinct protective functions.^1^^,^^2^ Non-glandular trichomes act as physical barriers, deterring herbivores, reflecting UV radiation, and reducing water loss, whereas glandular trichomes secrete defensive compounds (e.g., resins or toxins) or attractants to aid pollination.^3^^,^^4^

Selective pressures drive the evolution of multifunctional morphological traits, where a single structural feature frequently fulfills multiple adaptive roles.^5^^,^^6^ Classic examples include cacti, whose spines defend against herbivores while also providing shade to reduce stem temperature and water loss.^7^^,^^8^ Trichomes are not exceptional in this sense:^9^ on young leaves of *Verbascum thapsus*, they deter grasshopper herbivory and reduce transpiration under high temperatures.^10^

**Figure 1.**
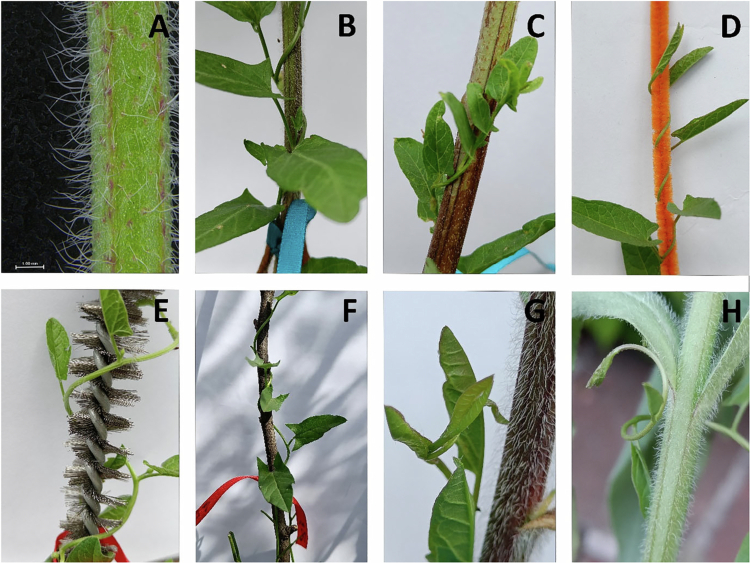
Non-glandular trichomes on *A. officinalis* stem (A); coiling of *C. arvensis* stem in *Anchusa* intact treatment (B); *Anchusa* without trichomes (C), artificial soft (D) and hard (E) trichome treatment, wooden stick treatment (F); initial contact of growing *C. arvensis* with *A. officinalis* (G); observed reduced coiling interaction of *C. arvensis* with *A. officinalis* stems (H).

The trichomes on *Anchusa officinalis* L. are non-glandular and primarily protect the plant against water loss^11^ (Figure 1A). This species coexists with climbing plants, which exhibit increased reproductive success when growing on hosts than free-standing individuals,^12^ as hosts provide structural support that enhances access to light and pollinators.^13^ Twining species, which rely on thigmotropism to coil around hosts,^14^^,^^15^ show reduced colonization on *A. officinalis*, possibly due to its trichome-covered surfaces that may interfere with mechanically sensitive climbers. Thus, I hypothesize that the non-glandular trichomes on *A.officinalis* stems serve as a defense against the climbing plant *Convolvulus arvensis* L., a frequently coexisting species.

## Methods

### Study species

Field bindweed (*C. arvensis*, Convolvulaceae) is a common perennial weed that climbs on neighboring plants and fences. It is native to Europe and Asia and grows in a wide range of conditions, particularly in open, disturbed areas. *C. arvensis* has deep, persistent roots and slender, twining stems that can stretch anywhere from 20 cm to 2 m in length.^16^ These flexible stems coil tightly around nearby supports, allowing the plants to climb efficiently. Like many twining vines, its shoot tips perform a slow circular motion called circumnutation, a circular movement which enhances the plant's ability to encounter and coil around nearby structures.^17^ Unlike climbing plants that use tendrils or sticky roots for attachment, *C. arvensis* depends entirely on its spiraling stems to hold itself upright. This simple but effective strategy lets it dominate surrounding vegetation, often outcompeting other plants for light and space. Flowers are insect-dependent and self-incompatible. Most plants in Slovakia bloom between June and September (pers. obs.). Flowers are predominantly visited by Hymenoptera, Lepidoptera, and Diptera. The number of seeds per capsule varies between one and four.^18-22^

The common bugloss (*A. officinalis*, Boraginaceae) is a common perennial weed native to Europe and some parts of Asia. Similar to *C. arvensis*, it occupies open, disturbed habitats such as roadsides, fields, and ditches. Flowers are insect-dependent, pollinated predominantly by bumblebees, and self-incompatible. It blooms between May and September of each year. It produces up to four seeds per nutlet.^11^^,^^23^^,^^24^

### Field survey

I surveyed an urban plot (48°22′39.0"*N*, 17°35′36.2"E) densely occupied by both *C. arvensis* and *A. officinalis* on June 2, 2025. I counted all *A. officinalis* stems with *C. arvensis* nearby. I recorded whether *A. officinalis* grew with or without coiled *C. arvensis*.

### The experiment

*C. arvensis* (*n* = 120) and *A. officinalis* (*n* = 50) plants were collected from abandoned fields near Trnava, Slovakia (48°22′38.7"*N*, 17°35′14"E), during April 2025 and transplanted into pots (0.4 l and 2 l, respectively) filled with standard gardening substrate and drainage. Due to the low germination rate observed in *C. arvensis* seedlings, only established plants were used in the experiment. The potted plants were maintained in a humid substrate and placed outdoors in a natural environment on private property for four weeks, ensuring that they were not exposed to direct sunlight but received ambient light and humidity. These conditions were maintained until the end of the experiment in June 2025.

### Experimental procedure

Convolvulus plants were randomly assigned to one of four treatments (*n* = 21 per treatment): Anchusa intact, trichomes removed, artificial soft trichomes, artificial hard trichomes and wooden sticks (Figure 1). Trichomes were removed from *A. officinalis* stems in the second treatment using a Swedish dishcloth. I initially attempted to remove trichomes using a paintbrush^25^ but found this method insufficient for complete removal. The removal of trichomes did not harm the plants, as they generally continued flowering at least until the end of the experiment. For the artificial trichome treatments (Figure 1B–D), I used commercially available wire rods equipped with either soft plastic bristles or hard steel bristles.

The *C. arvensis* stem was gently attached approximately 2 cm below its terminal growth point to the respective coiling support (e.g., *Anchusa* intact, wooden stick) using a numbered ribbon. The width of the coiling support was measured using digital calipers. During attachment, care was taken to ensure that all *C. arvensis* individuals, a left-coiling species, were wrapped in a leftward direction to maintain consistency. All treatments were performed simultaneously, except for the artificial hard trichome treatment, which was conducted in June 2025. Stem coiling occurrence and number were recorded every 24 h, scored to the nearest 0.5 coil, over a period of 7 d. The speed of coiling was defined as the number of days until *C. arvensis* coiled fully for the first time around the host plant (or any other experimental object).

### Statistical analyses

The likelihood of *C. arvensis* coiling (or not) around *A. officinalis* in the field was examined using a binomial test. Spearman's rank correlation coefficients was caculated for possible association between the width of coiling support and coiling speed/number for each treatment except for the Artificial soft and hard trichomes treatments, because the width of soft and hard wire rods was almost uniform (5.43 mm, SD = 0.33, *n* = 6, 19.68 mm, SD = 1.0). Differences between treatments in the likelihood of *C. arvensis* coiling at least once around the coiling support were analyzed using logistic regression. Survival analysis was used to examine the time to the first full coil of *C. arvensis* around the coiling support (in days) across different treatments. This approach accounts for both the timing of coiling events and plants that never coiled during the observation period. Differences in the maximum number of coils per plant were analyzed using a generalized linear model with a negative binomial distribution of the data. Data were analyzed with The Jamovi Project.^26^

## Results

### Field survey

A total of 169 *A. officinalis* stems with *C. arvensis* nearby (i.e., close to the stem) were recorded in the study area. The majority of *C. arvensis* (163/169 96%) individuals were observed not to coil around *A. officinalis*. Most colied around *Elytrigia* sp. (*n* = 161), one on *Cichorium intybus* L., and one on *Galium mollugo* L.). This strong bias against coiling *A. officinalis* was highly significant (binomial test: *p* <  0.001).

### The experiment

#### Differences of coiling among treatments

**Figure 2.**
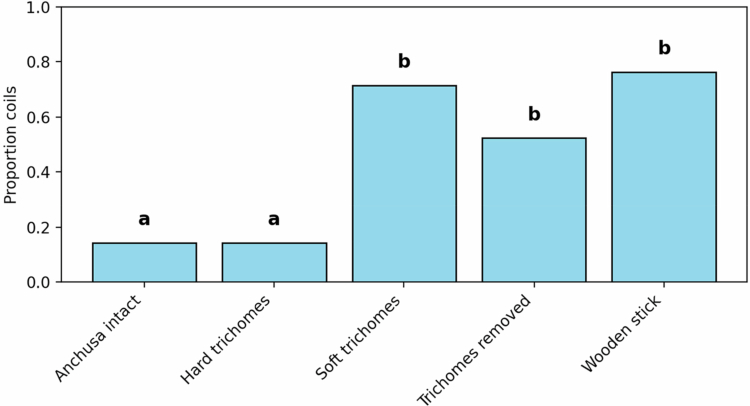
The proportion of *C. arvensis* coiled at least once around coiling support. Treatments with different letters are significantly different (*p* < 0.001).

Spearman's rank correlation coefficients for each treatment group showed no evidence of a relationship between coiling support width and coiling speed and number within each treatment group (all *p* > 0.4). *C. arvensis* in the *Anchusa* intact treatment (3/21, 14%) and hard trichomes treatment (3/21, 14%) coiled significantly less frequently than in the wooden stick (16/21, 76%) (Table 1), artificial soft trichome treatments (15/21, 71%) (Table 1), and with the trichomes removed treatment (11/21, 52%) (*p* = 0.039) (Figure 2). The differences between artificial soft trichomes, trichome removal, and wooden stick treatment were not significant. After initial contact with trichomes, *C. arvensis* stems were typically observed to change their growth direction, resulting in a lack of coiling around the *Anchusa* stem (Figure 1E, F).

**Table 1.** Logistic regression on the proportion of plants coiled by treatment.

Variable	Coefficient	SE	*z*	*p*	95% CI
Intercept	−1.792	0.624	−2.873	0.004	(−3.014, −0.570)
Artificial trichomes	2.708	0.789	3.433	<0.001	(1.162, 4.254)
Hard trichomes	−9.451e-16	0.882	−1.07e-15	1.0	(−1.729, 1.729)
Trichomes removed	1.887	0.761	2.478	0.013	(0.395, 3.379)
Wooden stick	2.955	0.807	3.661	<0.001	(1.373, 4.537)

#### Speed of coiling

**Figure 3.**
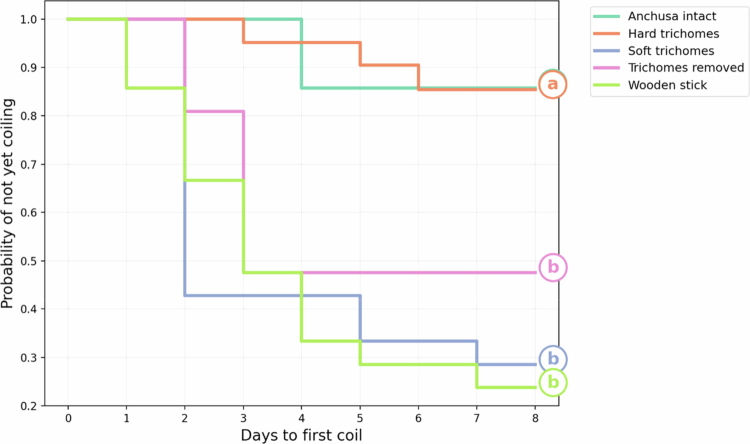
The difference in coiling speed among treatments. Treatments with different letters are significantly different (*p* < 0.05). *C. arvensis* in *Anchusa* intact treatment, and in hard trichomes treatment, started to coil significantly later than plants in the remaining three treatments.

There were significant differences among the treatments (Log-rank Test: *χ*^2^ = 24.9, df = 4, *p* < 0.001, Figure 3). *Anchusa* intact differed from every other treatment (all *p* ≤ 0.005), confirming that intact *Anchusa* plants strongly inhibited coiling behavior. Hard trichomes did not differ from *Anchusa* intact (*p *= 0.998), indicating that a rigid trichome barrier likewise prevents rapid coiling. *C. arvensis* bearing soft trichomes showed the fastest response (median = 2 d), and wooden stick produced an equally rapid latency (median = 2 d); these two treatments did not differ (*p *= 0.72). Trichomes removed displayed an intermediate response (median = 3 d), faster than *Anchusa* intact and hard trichomes (*p *= 0.005 and *p *= 0.006, respectively) but slower than the two quickest treatments (*p *≥ 0.12). The median was not calculated for *Anchusa* intact and hard trichomes because most plants in these groups did not coil within the 7-d observation period.

#### Number of coils

**Figure 4.**
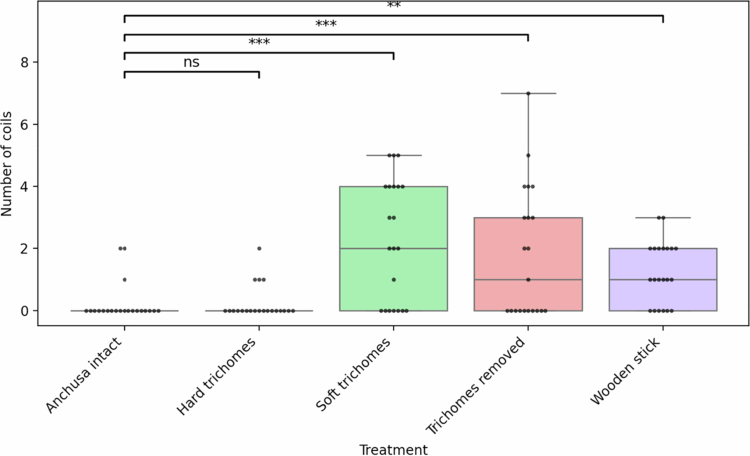
Differences in number of coils among treatments. The colored boxes show the interquartile range (IQR), the horizontal line inside each box is the median, whiskers extend to 1.5 × IQR beyond the 1st and 3rd quartiles, and individual-colored dots represent all data points with slight horizontal jitter for visibility. Black brackets with ** and *** indicate statistically significant differences (*p *< 0.01 and <0.001, respectively), while “ns” shows non-significant comparisons.

There were significant differences in the number of coils (±0.5) among the five treatments (LR *χ*^2^ = 37.2, df = 4, *p *< 0.0001; Figure 4, Table 2). Intact *Anchusa* plants strongly suppressed coiling, and the hard-trichome mimic produced an indistinguishable response (*p *= 0.998). By contrast, soft trichomes, trichomes removed, and wooden stick each produced a 3- to 6-fold increase in coil formation (all *p *< 0.0001 vs. *Anchusa* intact/hard trichomes). These three high-coil treatments did not differ from one another (all *p *≥ 0.12).

**Table 2.** Generalized linear model with negative binomial distribution on number of coils.

Variable	Coefficient	SE	*z*	*p*	95% CI
Intercept	−1.4351	0.498	−2.884	0.004	(−2.41, −0.46)
Hard trichomes	2.79E-13	0.704	3.97E-13	1	(−1.379, 1.379)
Soft trichomes	2.2618	0.562	4.023	<0.001	(1.16, 3.364)
Trichomes removed	2.0281	0.567	3.577	<0.001	(0.917, 3.14)
Wooden stick	1.6487	0.578	2.854	0.004	(0.516, 2.781)

## Discussion

This study found that *A. officinalis* can inhibit the coiling behavior of *C. arvensis*, a common parasitic climber. In natural settings, *C. arvensis* stems rarely coiled around *A. officinalis*, and all experimental treatments, except for the artificial hard trichomes, resulted in a similar increase in coiling activity.

Although the non-glandular trichomes of *A. officinalis* are typically thought to help reduce water loss in dry, sunny environments,^11^^,^^24^ their primary function may not be related to herbivore deterrence. *C. arvensis*, one of the world’s most destructive agricultural weeds, damages host plants mainly through its aggressive climbing, which increases competition for resources and can physically damage crops.^27^^,^^28^ By monopolizing light, water, and nutrients, *C. arvensis* can cause severe yield losses, especially in crops such as maize and wheat.^29^^,^^30^ In this context, any structural feature, such as trichomes, that helps a plant resist climbing could offer a significant ecological advantage.

The exact factors underlying the low frequency of coiling by *C. arvensis* around *A. officinalis* remain unclear. One possibility is biochemical deterrence; although trichomes are usually considered physical barriers, some studies suggest that they may also secrete compounds that repel other organisms or influence their behavior.^1-3^ In some plants, even non-glandular trichomes have been linked to the release of bioactive chemicals.^31^In our study, *C. arvensis* coiled more quickly and frequently around stems where trichomes had been removed compared to intact *A. officinalis*. This does not suggest that chemical residues remaining on the stem may continue to influence climbing plant attachment even after the trichomes are removed. However, my measurements cannot single out the possible chemosensoric mechanisms involved in coiling around the host plant size coding of plants; therefore, further research is needed to clarify this aspect.

Another explanation is mechanical: according to the thigmotropic response hypothesis *C. arvensis* detects and responds to the touch of trichomes, which could inhibit coiling. As shown in Figure 1G, H, when a *C. arvensis* stem touches a surface, it may 'probe' the substrate before committing to coiling. The plant might retract or grow away if the trichomes are too sharp. This was explanation was confirmed in the hard trichomes treatment. Although a reduced frequency of coiling around intact *A. officinalis* was observed, direct evidence that *C. arvensis* responds to touch cues via thigmotropism remains lacking.

Studies on *Pisum* *sativum* tendril twining movements by Guerra et al.^32^ and Ceccarini et al.^33^ proposed additional mechanisms that may enable plants to assess support properties. One possibility involves echolocation, where plants produce sonic clicks and analyze the returning echoes to gather environmental information.^34^ This biological sonar system could potentially inform plants about the characteristics of support surfaces, enabling appropriate responses. Another proposed mechanism involves ocelli-like structures within the upper and subepidermal leaf cells, which may function as primitive visual sensors for environmental assessment.^35^ While I lack direct evidence for either mechanism, the substrate ‘probing’ behavior observed in Figure 1G, H provides some support for these hypotheses.

Interestingly, sharp, non-glandular trichomes have been observed in other plants, such as rice (*Oryza sativa* cv. Nipponbare) can puncture insect guts and offer a strong defense.^36^ It is possible that the stiff trichomes of *A. officinalis* create similar mechanical stress on *C. arvensis* stems, which might trigger internal signaling (such as calcium influx) that disrupts growth.^15^^,^^37^ However, if the effect is purely mechanical, then why does *C. arvensis* coil more readily around artificial soft trichomes?

The answer may lie in the properties of the artificial materials: the soft, flexible artificial trichomes likely did not replicate the stiffness or sharpness of natural ones. This softness may have allowed *C. arvensis* to compress and grip the underlying stick easily, closely resembling its preferred support structures in nature, such as grass-like stems. The present experiment showed that hard artificial trichomes are very effective in preventing coiling.

One possible shortcoming in the present experiment is that the artificial trichomes (soft and hard) did not have identical diameters. For instance, tendrils of **P*. sativum* showed shorter movement time for a thinner stimulus than for a thicker one, suggesting that the diameter of the supportive object plays an important role.^32^^,^^33^ It is, however, highly improbable that the wider diameter of hard trichomes could significantly influence the results. First, *C. arvensis* can coil around substrates wider than 21 mm in diameter (*P*. Prokop, pers. obs.). Second, the growth rate of *C. arvensis* was sufficiently high during the 7-d observation period, imposing no constraints on coiling activity. Therefore, hard trichomes strongly suppressed coiling independently of their diameter.

Overall, this study broadens our understanding of the functions of non-glandular trichomes. In addition to their established roles in reducing herbivory, conserving water, and reflecting UV light,^1^^,^^2^ I propose a new function for trichomes: reducing the attachment of climbing plants. These results support the idea that plant structures often serve multiple ecological roles.^7-10^ It is also possible that these results are not limited to trichomes but also to spinose structures, such as thorns, spines, and prickles.

Future work should explore the ecological trade-offs of using trichomes to block climbers and examine how features such as trichome density, size, or stiffness influence their effectiveness as a defense mechanism.

## Data Availability

Data are available online in the Supplementary Information section at the end of the article.

## References

[1] Karabourniotis G, Liakopoulos G, Nikolopoulos D, Bresta P. Protective and defensive roles of non-glandular trichomes against multiple stresses: structure–function coordination. J For Res. 2020;31(1):1–12. doi: 10.1007/s11676-019-01034-4

[2] Schuurink R, Tissier A. Glandular trichomes: micro-organs with model statusff. New Phytol. 2020;225(6):2251–2266. doi: 10.1111/nph.16283 31651036

[3] Han G, Li Y, Yang Z, Wang C, Zhang Y, Wang B. 2022. Molecular mechanisms of plant trichome development. Front Plant Sci. 13:910228. doi: 10.3389/fpls.2022.910228. 35720574 PMC9198495

[4] Wang X, Shen C, Meng P, Tan G, Lv L. Analysis and review of trichomes in plants. BMC Plant Biol. 2021;21:1–11. doi: 10.1186/s12870-021-02840-x 33526015 PMC7852143

[5] Arnold SJ. Morphology, performance, and fitness. Am Zool. 1983;23(2):347–361. doi: 10.1093/icb/23.2.347

[6] Friedman NR, Miller ET, Ball JR, Kasuga H, Remeš V, Economo EP. Evolution of a multifunctional trait: shared effects of foraging ecology and thermoregulation on beak morphology, with consequences for song evolution. Proc Biol Sci. 2019;286(1917) 20192474. doi: 10.1098/rspb.2019.2474 31847778 PMC6939928

[7] Mauseth JD. Structure-function relationships in highly modified shoots of cactaceae. Ann Bot. 2006;98(5):901–926. doi: 10.1093/aob/mcl133 16820405 PMC2803597

[8] Nobe PS. Environmental biology of agaves and cacti. Cambridge (UK): Cambridge University Press; 2003.

[9] Johnso NM, Baucom RS. The double life of trichomes: understanding their dual role in herbivory and herbicide resistance. Evolution. 2024;78(6):1121–1132. doi: 10.1093/evolut/qpae048 38518120

[10] Woodman RL, Fernandes GW. Differential mechanical defense: herbivory, evapotranspiration, and leaf-hairs. Oikos. 1991;60(1):11–19. doi: 10.2307/3544986

[11] Selvi F, Bigazzi M. Revision of genus *Anchusa* (Boraginaceae-Boragineae) in Greece. Bot J Linn Soc. 2003;142(4):431–454. doi: 10.1046/j.1095-8339.2003.00206.x

[12] Gianoli E. The behavioural ecology of climbing plants. AoB Plants. 2015;7:plv013. doi: 10.1093/aobpla/plv013 25678588 PMC4363473

[13] Heide-Jørgensen H. Parasitic flowering plants. Leiden (The Netherlands): Koninklijke Brill NV; 2008.

[14] Jaffe MJ. Thigmomorphogenesis: the response of plant growth and development to mechanical stimulation: with special reference to *Bryonia dioica*. Planta. 1973;114(2):143–157. doi: 10.1007/BF00387472 24458719

[15] Scorza LCT, Dornelas MC. Plants on the move: towards common mechanisms governing mechanically-induced plant movements. Plant Signal Behav. 2011;6(12):1979–1986. doi: 10.4161/psb.6.12.18192 22231201 PMC3337191

[16] Zouhar K. *Convolvulus arvensis*. In: Fire effects information system [Internet]. U.S. Department of Agriculture, Forest Service, Rocky Mountain Research Station, Fire Sciences Laboratory (Producer); 2004 [cited Aug 4, 2025]. Available from: https://www.fs.usda.gov/database/feis/plants/vine/conarv/all.html.

[17] Darwin C. The movements and habits of climbing plants. London (UK): John Murray; 1875.

[18] Mulligan GA. Autogamy, allogamy, and pollination in some Canadian weeds. Can J Bot. 1972;50(8):1767–1771. doi: 10.1139/b72-219

[19] Mulligan GA, Findlay JN. Reproductive systems and colonization in Canadian weeds. Can J Bot. 1970;48(5):859–860. doi: 10.1139/b70-119

[20] Prokop, P. 2024. Urban environment decreases pollinator availability, fertility, and prolongs anthesis in the field bindweed (*Convolvulus arvensis* Linnaeus, 1753). Plant Signal Behav. 19(1), 2325225. doi: 10.1080/15592324.2024.2325225 38448395 PMC10936644

[21] Prokop P, Neupauerová D. Flower closure in the field bindweed (*Convolvulus arvensis*): a field test of the pollination hypothesis. Turk J Bot. 2014;38(5):877–882. doi: 10.3906/bot-1310-57

[22] Waddington KD. Foraging patterns of halictid bees at flowers of *Convolvulus arvensis*. Psyche. 1976;83(1):112–119. doi: 10.1155/1976/45208

[23] Andersson S. Size-dependent pollination efficiency in *Anchusa officinalis* (*Boraginaceae*): causes and consequences. Oecologia. 1988;76(1):125–130. doi: 10.1007/BF00379610 28312389

[24] Chwil M, Weryszko-Chmielewska E. The structure of floral elements of *Anchusa officinalis* L. creating attractants for insects. Acta Agrobot. 2009;62(1):37–47. doi: 10.5586/aa.2009.005

[25] Zhang X, Oppenheimer DG. A simple and efficient method for isolating trichomes for downstream analyses. Plant Cell Physiol. 2004;45(2):221–224. doi: 10.1093/pcp/pch016 14988492

[26] The Jamovi Project. jamovi (Version 2.5) [computer software]. 2024. Available from: https://www.jamovi.org.

[27] Holm LG, Plucknett DL, Pancho JV, Herberger JP. The world's worst weeds: distribution and biology. Honolulu (HI): University Press of Hawaii; 1977.

[28] Phillips WM. Field bindweed and its control. In USDA Leaflet No. 476. 1967. pp. 1–7.

[29] Culhavi C, Manea DN. Controlling the perennial species *Convolvulus Arvensis* L., a problem-weed in winter wheat. Res J Agric Sci. 2010;42(4):32–37.

[30] Rusu T, Gus P, Bogdan I, Paulette L, Oroian I. Research regarding control of *Convolvulus arvensis* L. in relation to soil tillage systems. J Cent Eur Agric. 2006;7(4):739–742.

[31] Santos Tozin LRD, de Melo Silva SC, Rodrigues TM. Non-glandular trichomes in Lamiaceae and Verbenaceae species: morphological and histochemical features indicate more than physical protection. N Z J Bot. 2016;54(4):446–457. doi: 10.1080/0028825X.2016.1205107

[32] Guerra S, Peressotti A, Peressotti F, Bulgheroni M, Baccinelli W, D'Amico E, Gómez A, Massaccesi S, Ceccarini F, Castiello U. Flexible control of movement in plants. Sci Rep. 2019;9(1):1–9. doi: 10.1038/s41598-019-53118-0 31719580 PMC6851115

[33] Ceccarini F, Guerra S, Peressotti A, Peressotti F, Bulgheroni M, Baccinelli W, Bonato B, Castiello U. Speed–accuracy trade-off in plants. Psychon Bull Rev. 2020;27(5):966–973. doi: 10.3758/s13423-020-01753-4 32542481

[34] Gagliano M, Renton M, Duvdevani N, Timmins M, Mancuso S. Out of sight but not out of mind: alternative means of communication in plants. PLoS One. 2012;7(5), e37382. doi: 10.1371/journal.pone.0037382 22629387 PMC3358309

[35] Baluška F, Mancuso S. Vision in plants via plant-specific ocelliff. Trends Plant Sci. 2016;21(9):727–730. doi: 10.1016/j.tplants.2016.07.008 27491517

[36] Kaur I, Kariyat RR. Eating barbed wire: direct and indirect defensive roles of non-glandular trichomes. Plant Cell Environ. 2020;43(9):2015–2018. doi: 10.1111/pce.13828 32562284

[37] Hamant O, Inoue D, Bouchez D, Dumais J, Mjolsness E. Are microtubules tension sensorsff. Nat Commun. 2019;10(1):2360. doi: 10.1038/s41467-019-10207-y. 31142740 PMC6541610

